# The Predictive Role of miRNAs in Hepatitis B Vaccine Response of Metabolic Dysfunction-Associated Steatotic Liver Disease Patients

**DOI:** 10.3390/v16111799

**Published:** 2024-11-20

**Authors:** Gamze Guney Eskiler, Oguz Karabay, Mukaddes Tozlu, Ayhan Aydin, Kaan Furkan Hamarat, Umut Alkurt, Asuman Deveci Ozkan, Yasemin Gunduz

**Affiliations:** 1 Department of Medical Biology, Faculty of Medicine, Sakarya University, 54290 Sakarya, Türkiye; deveci@sakarya.edu.tr; 2 Department of Infectious Diseases and Clinical Microbiology, Faculty of Medicine, Sakarya University, 54290 Sakarya, Türkiye; okarabay@sakarya.edu.tr; 3 Department of Gastroenterology, Faculty of Medicine, Sakarya University, 54290 Sakarya, Türkiye; mukaddestozlu@sakarya.edu.tr; 4 Department of Internal Sciences, Sakarya University Training and Research Hospital, 54290 Sakarya, Türkiye; doctorayhanaydin@gmail.com; 5 Faculty of Medicine, Sakarya University, 54290 Sakarya, Türkiye; hamaratkaanfurkan@gmail.com (K.F.H.); umutalkurt19@gmail.com (U.A.); 6 Department of Radiology, Faculty of Medicine, Sakarya University, 54290 Sakarya, Türkiye; ygunduz@sakarya.edu.tr

**Keywords:** metabolic dysfunction-associated steatotic liver disease, hepatitis B, hepatitis B vaccine response, miRNA

## Abstract

(1) Background: Metabolic dysfunction-associated steatotic liver disease (MASLD) is the most common chronic liver disease. Although the changes in the expression levels of microRNAs (miRNAs) in hepatitis B virus-related diseases have been evaluated, no study has evaluated the role of miRNAs in HBV vaccine response in MASLD patients. We aimed to determine the miRNA expression profile in MASLD patients according to HBV vaccine response. (2) Methods: Overall, 100 MASLD patients and 100 controls were included, and anti-HBs levels were measured after three doses of HBV vaccine administration. After collecting blood samples, 22 different miRNA expression profiles were analyzed by RT-PCR analysis, and changes in the expression levels of potential miRNAs were further verified in all study groups. (3) Results: The miR-146a expression level considerably increased in MASLD patients compared to the control group. Furthermore, miR-99a and miR-640 expression levels significantly increased in AntiHBs (−) healthy individuals. (4): Conclusions: miR-146a could be used as the diagnostic marker in MASLD patients. Furthermore, the miR-99a and miR-640 expression levels could predict hepatitis B vaccine response. However, validation studies are required to verify the biomarker potential of miRNAs within a more significant number of patients.

## 1. Introduction

Hepatitis B disease is a serious worldwide health problem leading to acute hepatitis and asymptomatic chronic infection to cirrhosis and hepatocellular carcinoma. Hepatitis B virus (HBV) is estimated to cause more than 500,000 deaths each year, with 50% of deaths due to liver cancer and 30% due to cirrhosis. In addition, 1.2 million people are newly infected with HBV, and more than 250 million people develop chronic HBV infection in 2022 [1,2].

Vaccination against HBV is used in many countries to achieve national immunity. Although recombinant hepatitis B vaccine has been proven to be safe and effective in protecting people against HBV infection, approximately 5–10% of healthy individuals do not develop a protective anti-HBs titer (10 mIU/mL) after a standard three-dose vaccination regimen, and these individuals remain at risk of HBV infection. In this context, HBsAg-negative individuals with an anti-HBs titer < 10 mIU/mL within 1–6 months after the standard immunization program are defined as HBV vaccine non-responders. It is suggested that various factors such as age, obesity, smoking and genetic factors play an essential role in HBV vaccine non-responders [3,4,5].

The role of genetic predispositions, including human leukocyte antigen (HLA) and MHC-II polymorphisms, in HBV vaccine response are examined in the literature. HLA and MHC-II are involved in the presentation of viral peptides to CD-4 T helper cells and are thus associated with the non-response of HBV vaccine. Additionally, polymorphisms in cytokines, cytokine receptors and Toll-like receptors, low cytokine release caused by Th cells or hypersensitivity in B cells, advanced age, chronic diseases and immunomodulatory drugs are associated with HBV vaccine response/responsiveness because they cause the suppression of immune response [3,6,7]. Nevertheless, the molecular mechanisms causing the non-response of the HBV vaccine are still not understood.

Chronic HBV infection is a significant cause of chronic liver disease. HBV infection causes liver complications such as fatty liver, cirrhosis, hepatocellular carcinoma and fibrosis. Metabolic dysfunction-associated steatotic liver disease (MASLD) is one of the most common causes of chronic liver disease. MASLD is a metabolic stress-induced liver injury closely related to insulin resistance and genetic predisposition. It includes a spectrum of liver damage such as simple steatosis, metabolic dysfunction-associated steatohepatitis (MASH), fibrosis and cirrhosis [1,8,9,10]. It is estimated that 29.6% of HBV patients worldwide have concurrent MASLD. Furthermore, in a meta-analysis of 4100 HBV patients, body mass index (BMI), obesity, moderate alcohol consumption, diabetes, high serum triglycerides and HBV viral load are determined as risk factors associated with advanced fibrosis in MASLD patients. On the other hand, in case-control studies conducted in different populations, there are conflicting results that MASLD is less common in patients with HBV infection [1,11,12]. In this context, further studies should be carried out to understand better the relationship between HBV risk and infection and their association with MASLD for effective treatment strategies in patients with chronic HBV infection. In addition, molecular mechanisms underlying HBV vaccine response should be further investigated in the high-risk group of patients (MASLD, primary biliary cirrhosis, primary sclerosing cholangitis, cirrhosis, hepatitis C, autoimmune hepatitis, etc.) due to them having a higher risk of HBV infection than a healthy person.

MicroRNA (miRNA) are non-coding RNA molecules found in all eukaryotic cells and regulate gene expression by binding to the untranslated region of target genes. As miRNAs have been used not only as biomarkers but also as therapeutic targets in recent years, changes in miRNA expression can be associated with drug resistance and/or vaccine response [13,14,15]. miRNAs play an important role in HBV pathogenesis through direct and indirect interactions with the viral genome or proteins. In addition to the expression profiles of miRNAs in different HBV-related diseases, some miRNAs are closely associated with the stage of liver complications. Therefore, the expression profiles of miRNAs can be changed in the HBV-infected state and are closely related to the viral life cycle and host damage. Therefore, the potential role of miRNAs as biomarkers in the early diagnosis and treatment of HBV-related liver complications has attracted significant attention [13,14,15].

In this context, it was aimed to determine the potential predictive role of changes in the miRNAs expression levels in MASLD patients and healthy individuals in terms of HBV vaccine responder/non-responder and to reveal the risk of MASLD development in especially HBV vaccine non-responders at the epigenetic level.

## 2. Materials and Methods

### 2.1. Study Population

The study included 100 patients with MASLD and 100 healthy controls. MASLD patients included evidence of hepatic steatosis (HS) by imaging or histology and without secondary causes of hepatic fat accumulation, such as significant alcohol consumption, long-term use of steatogenic drugs, or inherited disease. Clinical and demographic characteristics, age, gender, and baseline viral markers (HBsAg, anti-HBc IgG, anti-HBcIgM, anti-HBs, antiHCV, antiHIV) were listed. The healthy control group had the same average age group as the patients with MASLD and did not use any immunosuppressive agents, any immunosuppressive disease and diabetes, and no pregnancy. One month after the third dose of vaccine administration, AntiHBs levels < 10 IU were considered vaccine non-responders and AntiHB levels > 10 IU were accepted as vaccine responders. This study was approved by the Ethics Committee of Sakarya University (application number: 16214662/050.01.04/70458/171), and written consent was obtained from all patients and the healthy group included in this study.

### 2.2. Collection of Blood Samples and miRNA Screening

Venous blood samples were collected one month after the third dose of HB vaccine administration from 100 MASLD and 100 healthy individuals and stored at −80 °C until the experiments. An miRNeasy Kit (Qiagen GmbH, Hilden, Germany) was used following the appropriate kit protocol. The obtained miRNA pellet was dissolved in 20 µL ribonuclease-free water and stored at −80 °C until analysis. The concentrations of miRNAs were measured using a Qubit Spectrophotometer (Thermo Fisher Scientific Inc., Waltham, MA, USA). To assess the expression of 21 different miRNAs, cDNA was synthesized using a OneScript^®^ Plus cDNA Synthesis Kit (Applied Biological Materials Inc. (abm), Richmond, BC, Canada) in pooled samples of each group and analyzed by Step One Plus™ Real-Time PCR (Applied Biosystems, Invitrogen, Waltham, MA, USA). RNU6B was used to normalize the data. The data obtained were evaluated using the RT2 Profiler PCR Array Data Analysis program.

### 2.3. Validation of Candidate miRNAs

The miR-99a, miR-146a, and miR-640 expression levels were further quantitatively validated by the Step One Plus™ Real-Time PCR system (Applied Biosystems) according to the screening of miRNA results. RNU6B was used to normalize the data.

### 2.4. Statistical Analysis

All statistical analyses were performed using SPSS (version 22.0). After performing the Kolmogorov–Smirnov test, Mann–Whitney U and Kruskal–Wallis tests were used to determine the differences between groups. ROC curves were generated to determine the predictive importance of candidate miRNAs for MASLD, and cut-off values were determined by calculating the area under the ROC curves (AUC). In the study, *p*-values below 0.05 were considered statistically significant.

## 3. Results

### 3.1. Patient Data

This study included 100 MASLD patients and 100 controls. Blood samples were collected from 32 AntiHBs negative (−) and 68 AntiHBs positive (+) individuals in the control group. The MASLD group collected samples from 16 MASLD AntiHBs negative (−) and 84 MASLD AntiHBs positive (+) patients. For the whole study, Group 1: control group AntiHBs positive (+), Group 2: control group AntiHBs negative (−), Group 3: MASLD AntiHBs negative (−) patients and Group 4: MASLD AntiHBs positive (+) patients. The clinical data of the groups are summarized in Table 1. The control group consisted of 54 females and 46 males (Group 1: 37 females and 31 males, Group 2: 17 females and 15 males), and the MASLD group consisted of 66 female and 34 male participants (Group 3: 12 female and 4 male and Group 4: 54 female and 30 male). The median age of Group 1 and Group 2 was 44 (41–48) and 41.5 (37–51) years, respectively, while the median age of Group 3 and Group 4 was 59 (49–62) and 47 (44–51) years, respectively, and there was a statistically significant difference between groups (*p* = 0.01). Body mass index was analyzed as 24.69 (22.58–25.40), 27.93 (24.51–33.90), 33.38 (31.69–34.89) and 31.31 (29.75–32.35) in Group 1, Group 2, Group 3 and Group 4, respectively and a statistically significant difference was found between the groups (*p* = 0.000). In addition, there was a significant difference between glucose, ALT, AST, GGT, ALP, cholesterol, triglyceride, LDL, HOMA-IR, insulin and HbA1c values between healthy individuals and MASLD (*p* < 0.01).

When non-invasive fibrosis scores were analyzed, a significant difference was found between Group 3 and Group 4 in the NFS score (Table 2, *p* = 0.001). According to ISHAK fibrosis scoring, 85% of MASLD scores were 0–1, 13% were 2–3 and 2% were 4–6. According to MetaViR scoring, 57% were F0–F2, 9% were F3–F4 and 27% were indeterminate.

### 3.2. miRNA Screening Results

**Figure 1:**
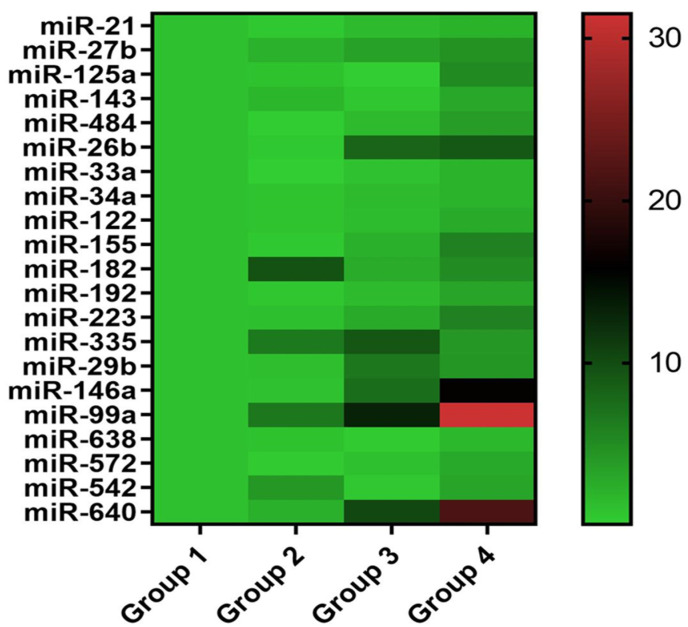
The screening of different miRNAs in MASLD patients and control group in terms of hepatitis B vaccine response (Group 1: control group AntiHBs positive (+), Group 2: control group AntiHBs negative (−), Group 3: MASLD AntiHBs negative (−) patients and Group 4: MASLD AntiHBs positive (+) patients).

The expression levels of different miRNAs were analyzed in four individuals (two females and two males) in each group, and the obtained results are summarized in Figure 1.

Although the expression levels of miR-21, miR-125a, miR-484, miR-26a, miR-33a, miR-34a, miR-122, miR-192, miR-638 and miR-572 in AntiHBs (−) healthy individuals (Group 2) compared to the AntiHBs (+) control group were downregulated, miR-182, miR-335 and miR-99a were significantly upregulated (*p* < 0.05). Furthermore, miR-125a, miR-143, miR-33a, miR-638, miR-542 were downregulated in AntiHBs (−) MASLD patients (Group 3) compared to the AntiHBs (+) control group, whereas especially miR-27b, miR-26b, miR-335, miR-29b, miR-146a, miR-99a and miR-640 expression levels were significantly upregulated. In AntiHBs (+) MASLD patients (Group 4), the expression levels of miR-27b, miR-125a, miR-26b, miR-155, miR-182, miR-223, miR-146a, miR-99a and miR-640 increased more than 5-fold.

### 3.3. Verification of Identified miRNA Alterations

**Figure 2:**
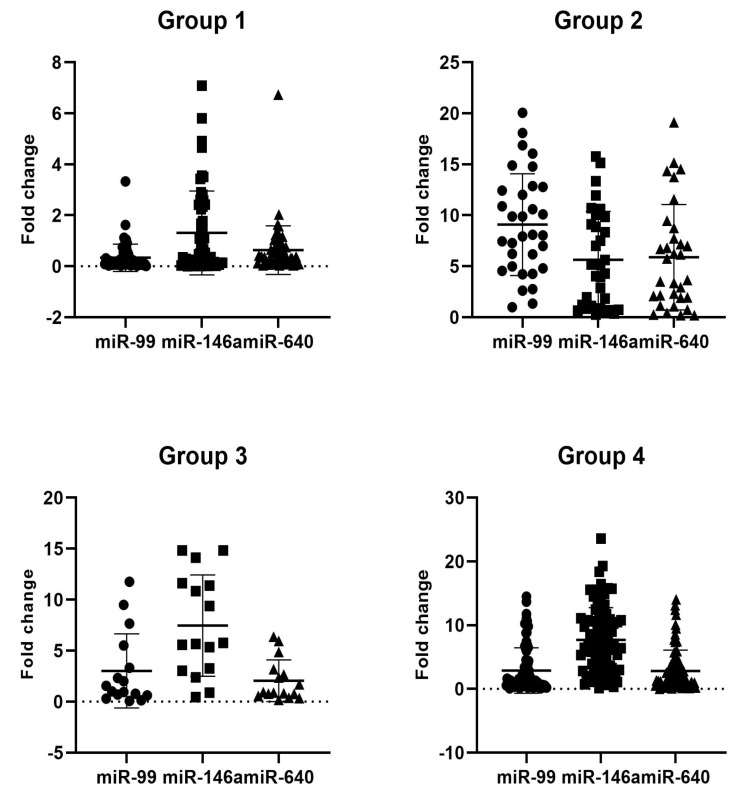
The expression of miR-99, miR-146a, and miR-640 was analyzed by RT-PCR in MASLD patients and the control groups (Group 1: control group AntiHBs positive (+), Group 2: control group AntiHBs negative (−), Group 3: MASLD AntiHBs negative (−) patients and Group 4: MASLD AntiHBs positive (+) patients).

According to the data summarized in Figure 1, miR-99a, miR-146a and miR-640, which were found to have a significant fold change (>5 fold) or group-specific alteration, were further analyzed in 100 MASLD patients and healthy controls. In Figure 2, miR-99a, miR-146, and miR-640 expression levels were analyzed as 7.46 (*p* = 0.00), 3.39 (*p* = 0.01), and 3.32 (*p* = 0.001), respectively, in AntiHBs (−) healthy individuals (Group 2) compared to the AntiHBs (+) control group. Furthermore, the level of miR-99a, miR-146, and miR-640 was 1.34 (*p* = 0.33), 5.29 (*p* = 0.003), and 1.24 (*p* = 0. 77), respectively, in AntiHBs (−) MASLD patients (Group 3) compared to the AntiHBs (+) control group, whereas 1.32 (*p* = 0.18), 5.57 (*p* = 0.001), and 1.46 (*p* = 0.063)-fold changes, respectively, were detected in AntiHBs (−) MASLD patients (Group 4).

### 3.4. The Relationship Between Changes in miRNA Expression and Clinical Parameters

Spearman correlation analysis was performed to determine the relationship between changes in miR-99, miR-146a, and miR-640 expression levels and glucose, ALT, AST, GGT, ALP, cholesterol, triglyceride, HDL, LDL, HOMA-IR, insulin and HbA1c values (Table 3 and Table 4). Although a statistically significant positive correlation was determined between the miR-99 expression level and HbA1c values, a significant negative correlation was determined between the miR-640 expression level and ALT (*p* < 0.05, Table 3). In Group 1, there was a statistically significant positive correlation between ALT and AST, GGT, and TG as well as AST-GGT, GGT-TG/LDL. In contrast, a significant negative correlation between GGT and HDL, as well as ALP with cholesterol levels, was found (*p* < 0.05, Table 3). In addition, statistically significant positive correlations were found between ALP and TG; cholesterol and TG/LDL/HbA1C; TG and LDL; LDL and HbA1c; HOMA-IR and insulin. In Group 2 healthy individuals, although a negative significant correlation was determined between the miR-99a expression level and AST; glucose and cholesterol/HDL; GGT and HDL; and HDL and Hb1Ac, a statistically positive significant correlation was analyzed between glucose and Hb1Ac; ALT and GGT/cholesterol/LDL; cholesterol and LDL; and HOMA-IR and Hb1Ac (Table 3).

In Group 3, a statistically significant positive correlation was analyzed between the miR-146 expression levels and Hb1ac values, although a negative significant correlation was analyzed between the miR-640 expression level and HOMA-IR values in MASLD patients (*p* < 0.05, Table 4). In addition, statistically significant positive correlations were determined between glucose and AST/Hb1AC; AST and GGT/FIB-4; cholesterol and LDL; and HOMA-IR and insulin/Hb1ac (*p* < 0.05). In Group 4, there was a significant positive correlation between the miR-99a expression level and TG, while a significant negative correlation was analyzed between the miR-640 expression level and NFS in MASLD patients (Table 4). In addition, statistically significant positive correlations were determined between glucose and GGT/cholesterol/HOMA-IR/Hb1AC/FIB-4/NFS; ALT and AST/GGT; AST and GGT/FIB-4; GGT and TG/FIB-4; cholesterol and TG/HDL/LDL; TG and LDL/Hb1AC; HOMA-IR and insulin; and insulin and FIB-4/NFS. On the other hand, significant negative correlations were analyzed between GGT and HDL; and cholesterol and HDL (*p* < 0.01, Table 4).

**Figure 3:**
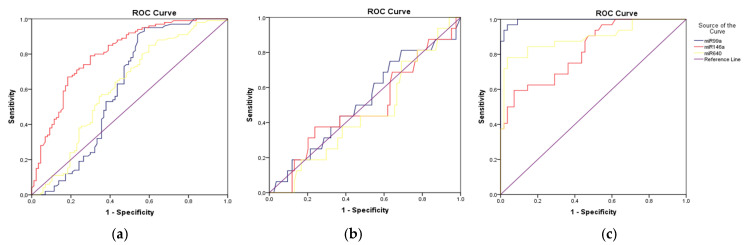
The ROC curve histograms of miRNAs in (**a**) MASLD/control group, (**b**) AntiHBs (−) MASLD/AntiHBs (+) MASLD and (**c**) AntiHBs (−) healthy individuals/AntiHBs (+) healthy individuals.

**Figure 4:**
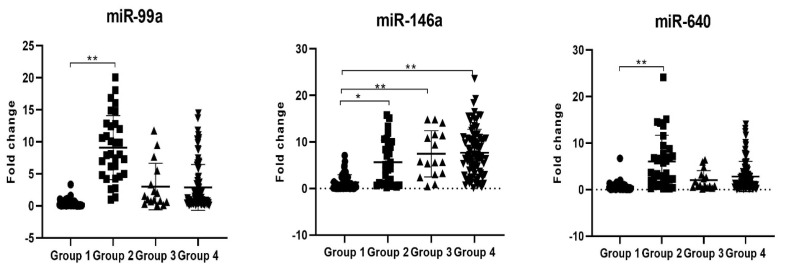
Comparison of miR-99a, miR-146a and miR-640 expression levels with groups according to ROC curve results (Group 1: Control group AntiHBs positive (+), Group 2: Control group AntiHBs negative (−), Group 3: MASLD AntiHBs negative (−) patients and Group 4: MASLD AntiHBs positive (+) patients) (*p* < 0.05 *, *p* < 0.01 **).

According to the results of ROC analysis (Figure 3), changes in miR-99a, miR-146 and miR-640 expression levels could significantly distinguish individuals diagnosed with MASLD from healthy individuals as diagnostic biomarkers (Figure 4), and the cut-off values were 0.88, 3.6, and 1.06, respectively (*p* < 0.01, Table 5). In addition, the discrimination of identified miRNAs in hepatitis B vaccine response in the MASLD group was insignificant (Table 6). On the other hand, miR-99a, miR-146a, miR-542 and miR-640 expression levels could significantly discriminate hepatitis B vaccine response in healthy individuals, and the cut-off values were analyzed as 1.24, 1.72, 1.10 and 1.05, respectively (Table 7).

## 4. Discussion

Herein, we first investigated the diagnostic and predictive role of miRNAs in hepatitis B vaccine response in MASLD patients compared to the control group. According to miRNA screening, there were >5-fold changes in the expression level of miR-99a, miR-146a and miR-640 in MASLD patients compared to the control group. Therefore, changes in miR-99a, miR-146a, and miR-640 expression levels were verified in 100 MASLD and healthy individuals. According to the findings, although the miR-146a expression level was significantly increased in individuals diagnosed with MASLD compared to the control group, miR-99a and miR-640 expression levels were significantly increased in AntiHBs (−) healthy individuals compared to AntiHBs (+) healthy people. On the other hand, miR-99a and miR-640 were downregulated in patients with MASLD compared to the control group. Additionally, especially miR-146a can significantly distinguish patients diagnosed with MASLD from healthy individuals as a diagnostic marker. Furthermore, the miR-99a and miR-640 expression levels as predictive biomarkers for hepatitis B vaccine response in healthy individuals were also revealed.

MASLD constitutes a significant proportion of liver diseases, is characterized by lipid accumulation in the liver, and is closely associated with mitochondrial abnormalities. Generally, abnormalities in mitochondrial function and increased reactive oxygen species affect fatty liver disease through insulin sensitivity. Increased ROS production increases the oxidation of fatty acids, leading to steatohepatitis and inflammation in the liver [11,12]. Nevertheless, the pathogenesis of MASLD is not yet clearly understood.

HBV infection is a global health problem affecting millions of people worldwide and causing chronic liver diseases, especially cirrhosis and hepatocellular carcinoma. It is reported that at least one third of cirrhosis patients and 75% of primary liver cancers are infected with HBV, and approximately 1 million people die each year from acute or chronic HBV infection [16]. The HBV genome has a double-chain circular DNA molecule and encodes four essential HBV proteins (C, X, P and S). Hepatitis B protein X (HBx) is a regulatory protein that plays multiple roles in cell signal transduction pathways and gene transcription. HBV infection and HBx expression affect many cell processes, such as cell division, cell cycle, and apoptosis. Therefore, there is likely a relationship between HBV infection and MASLD [12,17].

The hepatitis B vaccine is essential in reducing the mortality rate of HBV-related liver diseases as well as protecting against HBV. Only one study assesses the relationship between HBV vaccination and MASLD. The study of Joshi et al. (2021) states that HBV vaccine-specific immune responses are decreased in obese individuals with MASLD [10]. On the other hand, Roni et al. (2013) note that 52 cirrhotic liver disease patients have lower hepatitis B titers compared to the general population, and the response to HBV vaccination is lower, mainly due to age and disease progression [18].

Additionally, changes in different miRNA expression are associated with the development of MASLD [19,20,21,22,23,24,25]. In the study of Zhang et al. (2012), miR-122, miR-638, and miR-572 expression levels are different in patients with MASLD and chronic HBV, and thus, miRNAs could be a potential biomarker in the diagnosis of liver complications [20]. Wang et al. (2012) state that miR-122 expression is significantly decreased in liver biopsy samples in individuals with HBV infection, and the decreased expression of miR-122 is associated with increased HBV replication [21]. In a study by Roderburg et al. (2011), the expression levels of miR-29 family members decreased in fibrotic/cirrhotic tissues compared to non-fibrotic tissues [22]. Furthermore, miR-122 miR-22 and miR-99a expression levels are significantly increased in HBV-infected patients [23,24]. In a systematic review and meta-analysis, miR-34a, miR-122 and miR-192 can distinguish MASH from MASLD and may play a role as potential diagnostic biomarkers [25]. However, no study has evaluated the relationship between altered miRNA expression and HBV vaccine response in patients with MASLD or HBV infection. The current study evaluated changes in 22 different miRNA expression levels in AntiHBs (−) and AntiHBs (+) MASLD patients and healthy individuals. According to the findings, especially the miR-146a expression level was significantly upregulated in MASLD patients and could be used as a diagnostic biomarker. The overexpression of miR-146a causes the development of liver fibrosis due to inhibition of the Wnt signaling pathway and suppression of cell proliferation and thus may contribute to the progression of MASH [26]. Furthermore, exosomal miR-146a secreted into plasma may be a potential diagnostic and prognostic biomarker in diagnosing both hepatocellular carcinoma and cirrhosis [27]. In the study by Bandiera et al. (2016), miR-146a deregulates metabolic pathways associated with liver diseases and could be associated with HBV progression while inducing viral replication [28]. In this context, an increased miR-146 expression level could be a potential diagnostic biomarker for MASLD patients.

Furthermore, a limited number of studies have evaluated the roles of miRNAs in HB vaccine response in healthy individuals. Xiong et al. (2013) investigated the effect of single nucleotide changes (SNPs) in miR-146a, miR-196a, miR-27a, miR-26a-1, miR-124 and miR-149 genes on HBV vaccine response in healthy individuals. As a result of this study, specific SNPs in miRNA genes may affect the response to HBV vaccine, and there is a relationship between SNPs in miR-146a and miR-26a-1 genes and non-responders [29]. Additionally, increased miR-155 levels in serum play an important role in a non-response to the HBV vaccine in 1451 healthy individuals [30]. In the current study, we, for the first time, determined that miR-99a and miR-640 expression levels were significantly increased in AntiHBs (−) healthy individuals compared to AntiHBs (+) control and thus could distinguish vaccine response as a predictive biomarker for healthy individuals.

miR-99a is the sixth most abundant miRNA in healthy human liver, and a low expression of miR-99a is associated with poor prognosis in individuals diagnosed with hepatocellular carcinoma [31]. In addition, changes in the expression level of miR-99a are inversely correlated with GGT levels in individuals with MASH [32]. Xiong et al. (2016) state that miR-99a may be a potential biomarker in the chronic hepatitis B stage of liver disease and in the early diagnosis of HBV-associated hepatocellular carcinoma [33]. On the other hand, the miR-640 expression level is upregulated in HCV-positive individuals after hepatocellular carcinoma [34]. Furthermore, miR-640 suppresses the Wnt signaling pathway, leading to inflammation in Kuppfer cells and thus causing acute liver injury [35]. In this context, decreased miR-99a and miR-640 expression levels in AntiHBs (−) and AntiHBs (+) MASLD patients compared to AntiHBs (+) healthy individuals supported the relevant literature. However, changes in these miRNAs expression did not distinguish hepatitis B vaccine response. Therefore, further studies should be performed to assess changes in the expression of these miRNAs in MASLD patients.

Finally, the pathogenesis of MASLD is closely related to obesity, type 2 diabetes mellitus and insulin resistance and leads to the development of hepatic steatosis, fibrosis and cirrhosis. In the literature, the risk of MASLD development is 5-fold higher in diabetic patients with insulin resistance compared to normal individuals [36,37]. In the present study, a negative correlation between miR-640 expression level and HOMA-IR value and a statistically significant positive correlation between miR-146/miR-542 expression levels and Hb1ac were analyzed in AntiHBs (−) MASLD patients. Overall, 36 of the 100 MASLD patients had diabetes mellitus. In this framework, changes in the expression levels of these miRNAs could be associated with signaling pathways causing insulin resistance and could indicate biomarker potential in determining the risk of MASLD development in diabetic patients. However, miRNA expression levels should be investigated in a more comprehensive patient group, considering diabetes status.

## 5. Conclusions

In conclusion, our results suggest that changes in miRNA expression could have diagnostic and predictive potential in AntiHBs (+) and AntiHBs (−) MASLD patients and AntiHBs (−) healthy individuals compared to AntiHBS (+) healthy individuals. According to the obtained data, increased miR-146a levels, as well as decreased miR-99a and miR-640 expression levels, could have diagnostic potential in MASLD patients. On the other hand, changes in miR-99a and miR-640 expression levels may have predictive biomarker potential in vaccine response in healthy individuals. However, validation studies with transcriptomic analysis should be performed in more comprehensive MASLD patient groups. Additionally, we especially analyzed the expression of miRNA in the blood of samples in contrast to liver biopsy samples due to the diagnostic or predictive potential of miRNAs with the minimally invasive protocol.

## Figures and Tables

**Table 1:** Demographic and clinical parameters of MASLD and control groups (Group 1: control group AntiHBs positive (+), Group 2: control group AntiHBs negative (−), Group 3: MASLD AntiHBs negative (−) patients and Group 4: MASLD AntiHBs positive (+) patients).

	Group 1		Group 2		Group 3		Group 4		*p*
	Median	95% CI	Median	95% CI	Median	95% CI	Median	95% CI	
Age	44	41–48	41.5	37–51	59	49–62	47	44–51	0.01
BMI (kg/m^2^)	24.69	22.58–25.40	27.93	24.51–33.90	33.38	31.69–34.89	31.31	29.75–32.35	0.000
Hemoglobin	14	13.10–14.60	13.60	13.10–14.90	13.25	12.80–14.30	14.15	13.70–14.70	0.265
Glucose (mg/dL)	96	93–99	106	98–113.62	114.5	98–152	105	102–116	0.000
ALT (U/L)	17	15–19	19	14–26	22	15–32	27	23–36	0.000
AST (U/L)	19	17–21	17	16–19	19.5	17–27	24	21–26	0.000
GGT (U/L)	16	15–19	20	16–26	31.5	20–64	27.5	22–39	0.000
ALP	76	68–78	73.5	63–82	90	68–109	80	72–86	0.033
Albumin (g/L)	42.70	41.80–43.40	41.85	39.60–44.90	40.05	36.50–44.60	42.85	40.80–44.20	0.369
Total protein (mg/dL)	70.10	69.20–71.20	72	70.00–73.50	69.45	67.30–74.60	72.20	70.90–73.80	0.257
Cholesterol (mg/dL)	191	184–204	199.50	177–209	174.50	153–224	208.50	204–216	0.015
Triglyceride (mg/dL)	125	104–152	138.50	101–172	126	101–216	170	145–184	0.007
HDL (mg/dL)	47	43–52	48.50	44–50	39	35–54	48	44–51	0.477
LDL/(mg/dL)	121	112–131	137	126–153	123.5	103–158	142	135–150	0.004
HOMA-IR	1.63	1.30–1.92	2.94	2.14–5.57	5.38	4.21–9.29	3.74	2.96–4.56	0.000
Total bilirubin	0.60	0.50–0.70	0.50	0.40–0.65	0.61	0.40–0.90	0.58	0.50–0.65	0.470
Direct bilirubin	0.10	0.09–0.12	0.10	0.08–0.13	0.12	0.07–0.13	0.09	0.08–0.10	0.311
Urea	24	21–26	26	22–28	26	22–31	26	23–29	0.332
Creatinine	0.65	0.61–0.71	0.72	0.58–0.81	0.65	0.53–0.74	0.68	0.61–0.70	0.794
Insulin	6.70	5.40–7.80	12.50	8.18–34.94	14.94	12.90–31.11	13.33	11.60–16.24	0.000
HbA1c	5.10	5.10–5.20	5.50	5.20–5.90	6.60	5.90–7.90	5.40	5.30–5.60	0.000

**Table 2:** NFS and FIB-4 scores of Group 3 and Group 4 MASLD patients.

Parameter	Group 3		Group 4		*p*
	Median	95% CI	Median	95% CI	
NAFLD Fibrosis Score (NFS)	1.01	0.17–3.47	0.96	0.00–1.69	0.001
FIB-4 Score	1.12	0.64–2.07	0.83	0.74–0.94	0.079

**Table 3:** The relationship between changes in the expression level of miRNAs and clinical parameters in Group 1 and Group 2 healthy individuals (*p* < 0.05 *, *p* < 0.01 **).

		Glucose		ALT		AST		GGT		ALP		TC		TG		HDL		LDL		HOMA-IR		Insulin		HbA1c	
		−	+	−	+	−	+	−	+	−	+	−	+	−	+	−	+	−	+	−	+	−	+	−	+
miR99a	r	−00.119	0.070	0.206	−0.067	−0.436 *	0.201	0.208	−0.012	0.287	0.024	0.294	0.108	0.032	−0.064	−0.052	0.206	0.160	−0.006	0.643	0.041	0.143	−0.023	0.306	0.273 *
	*p*	0.524	0.609	0.266	0.625	0.014	0.141	0.262	0.928	0.125	0.864	0.115	0.431	0.867	0.644	0.785	0.131	0.399	0.965	0.119	0.768	0.736	0.868	0.129	0.046
miR146a	r	0.002	−0.122	−0.012	−0.045	−0.233	−0.109	0.235	−0.160	0.330	−0.147	0.079	−0.080	0.058	−0.068	−0.102	0.018	−0.068	−0.061	0.393	−0.048	0.643	0.058	0.228	−0.066
	*p*	0.993	0.376	0.950	0.745	0.207	0.426	0.204	0.242	0.075	0.285	0.676	0.560	0.761	0.621	0.593	0.894	0.722	0.656	0.383	0.732	0.086	0.675	0.262	0.635
miR640	r	−0.073	0.007	0.047	−0.372 **	0.150	−0.231	0.109	−0.245	−0.073	0.213	0.052	0.047	−0.223	−0.046	−0.125	0.192	0.152	−0.207	0.071	0.199	−0.048	0.122	0.087	0.070
	*p*	0.698	0.960	0.802	0.005	0.421	0.090	0.559	0.071	0.702	0.119	0.783	0.734	0.235	0.736	0.512	0.160	0.422	0.130	0.879	0.150	0.911	0.380	0.673	0.617
Glucose	r	1	1	0.088	−0.029	−0.009	0.107	0.264	0.078	−0.144	0.101	−0.369 *	−0.014	0.268	0.051	−0.378 *	−0.042	−0.139	−0.032	0.378	0.165	−0.084	0.041	0.438 *	−0.044
	*p*			0.638	0.835	0.961	0.435	0.151	0.573	0.449	0.461	0.045	0.919	0.152	0.713	0.039	0.763	0.462	0.818	0.403	0.234	0.844	0.767	0.025	0.754
ALT	r			1	1	0.326	0.639 **	0.475 **	0.678 **	−0.068	0.102	0.370 *	0.211	0.385 *	0.310 *	−0.278	−0.319 *	0.397 *	0.237	0.000	0.041	−0.240	0.128	−0.052	0.240
	*p*					0.073	0.000	0.007	0.000	0.720	0.458	0.044	0.123	0.036	0.021	0.137	0.017	0.030	0.082	1	0.770	0.568	0.356	0.801	0.080
AST	r					1	1	0.076	0.454 **	0.141	0.070	0.022	0.140	0.192	0.094	−0.047	−0.161	0.070	0.075	−0.613	0.063	−0.171	0.114	−0.250	0.250
	*p*							0.683	0.001	0.456	0.610	0.907	0.307	0.310	0.494	0.806	0.241	0.712	0.584	0.144	0.651	0.686	0.411	0.217	0.069
GGT	r							1	1	0.210	0.237	−0.194	0.288 *	0.303	0.438 **	−0.468 **	−0.347 **	−0.165	0.288 *	−0.143	0.113	−0.524	0.143	0.080	0.350 **
	*p*									0.266	0.082	0.305	0.033	0.103	0.001	0.009	0.009	0.383	0.033	0.760	0.415	0.183	0.303	0.699	0.010
ALP	r									1	1	0.087	0.325 *	−0.107	0.453 **	0.182	−0.088	−0.099	0.264	−0.429	−0.012	0.429	−0.095	−0.229	0.052
	*p*											0.653	0.016	0.579	0.001	0.344	0.522	0.610	0.051	0.337	0.929	0.289	0.494	0.270	0.710
TC	r											1	1	0.040	0.535 **	0.153	0.241	0.638 **	0.751 **	−0.571	−0.195	−0.119	−0.217	−0.117	0.351 **
	*p*													0.835	0.000	0.419	0.077	0.000	0.000	0.180	0.157	0.779	0.116	0.570	0.009
TG	r													1	1	−0.303	−0.247	0.250	0.410 **	−0.286	0.161	−0.452	0.101	0.291	0.180
	*p*															0.104	0.069	0.182	0.002	0.535	0.244	0.260	0.469	0.150	0.193
HDL	r															1	1	−0.118	0.133	−0.162	−0.214	0.252	−0.161	−0.396 *	−0.147
	*p*																	0.534	0.335	0.728	0.121	0.548	0.246	0.045	0.289
LDL	r																	1	1	0.127	−0.180	−0.434	−0.122	0.034	0.291 *
	*p*																			0.786	0.192	0.283	0.379	0.869	0.033
HOMA-IR	r																			1	1	0.750	0.892 **	0.775 *	−0.008
	*p*																					0.052	0.000	0.041	0.953
Insulin	r																					1	1	0.361	0.052
	*p*																							0.379	0.707
HbA1c	r																							1	1
	*p*																								

**Table 4:** The relationship between changes in the expression level of miRNAs and clinical parameters in Group 3 and Group 4 MASLD patients (*p* < 0.05 *, *p* < 0.01 **).

		Glucose		ALT		AST		GGT		ALP		TC		TG		HDL		LDL		HOMA-IR		Insulin		HbA1c		FIB-4		NAFLD	
		−	+	−	+	−	+	−	+	−	+	−	+	−	+	−	+	−	+	−	+	−	+	−	+	−	+	−	+
miR99a	r	0.099	−0.109	0.074	0.086	0.028	0.149	0.359	0.262 *	0.293	−0.006	−0.109	0.027	0.276	0.234 *	0.071	−0.046	−0.190	−0.049	0.028	−0.032	−0.192	−0.092	0.099	−0.017	−0.168	−0.039	−0.200	−0.085
	*p*	0.716	0.328	0.786	0.437	0.918	0.175	0.172	0.016	0.271	0.955	0.688	0.807	0.300	0.032	0.794	0.679	0.481	0.657	0.931	0.815	0.529	0.490	0.716	0.890	0.534	0.725	0.458	0.466
miR146a	r	0.330	0.042	0.246	−0.082	0.254	−0.062	0.450	0.018	0.203	0.135	0.221	0.045	0.127	0.176	0.115	−0.001	0.203	−0.020	0.098	0.124	−0.137	0.026	0.599 *	0.119	−0.087	−0.081	−0.074	0.008
	*p*	0.212	0.704	0.358	0.457	0.343	0.577	0.080	0.867	0.450	0.221	0.411	0.683	0.640	0.108	0.671	0.996	0.452	0.855	0.762	0.368	0.655	0.844	0.014	0.318	0.749	0.462	0.787	0.947
miR640	r	0.351	−0.154	0.412	0.123	0.312	0.147	0.232	0.124	0.146	0.154	0.235	0.211	0.088	0.137	0.050	0.162	0.321	0.178	−0.622 *	0.093	−0.533	0.114	0.220	−0.110	−0.106	−0.155	−0.147	−0.230 *
	*p*	0.183	0.163	0.112	0.266	0.240	0.181	0.387	0.260	0.590	0.162	0.380	0.054	0.745	0.215	0.854	0.140	0.226	0.107	0.031	0.499	0.061	0.394	0.414	0.355	0.696	0.160	0.587	0.045
Glucose	r	1	1	0.327	0.054	0.684 **	0.083	0.462	0.254 *	0.492	−0.020	−0.230	0.136	0.336	0.305 **	−0.124	−0.114	−0.181	0.201	0.256	0.292 *	−0.028	0.143	0.717 **	0.447 **	0.316	0.257 *	0.280	0.398 **
	*p*			0.216	0.626	0.003	0.457	0.071	0.020	0.053	0.854	0.392	0.220	0.204	0.005	0.647	0.304	0.503	0.070	0.422	0.030	0.929	0.288	0.002	0.000	0.233	0.019	0.294	0.000
ALT	r			1	1	0.772 **	0.877 **	0.527 *	0.615 **	0.315	0.043	0.037	0.030	0.078	0.196	0.123	−0.202	−0.049	0.062	0.007	0.139	−0.050	0.212	0.117	−0.019	−0.013	0.122	−0.047	−0.156
	*p*					0.000	0.000	0.036	0.000	0.234	0.701	0.892	0.788	0.774	0.075	0.651	0.065	0.858	0.575	0.983	0.312	0.872	0.110	0.665	0.874	0.963	0.271	0.862	0.178
AST	r					1	1	0.674 **	0.612 **	0.370	0.066	−0.188	0.049	0.112	0.207	−0.003	−0.148	−0.240	0.059	0.282	0.091	0.113	0.162	0.439	0.064	0.537 *	0.334 **	0.483	−0.093
	*p*							0.004	0.000	0.159	0.551	0.486	0.656	0.679	0.059	0.991	0.178	0.370	0.594	0.375	0.508	0.712	0.223	0.089	0.589	0.032	0.002	0.058	0.424
GGT	r							1	1	0.449	0.041	−0.338	0.095	0.171	0.400 **	−0.097	−0.285 **	−0.337	0.129	0.266	0.093	−0.082	0.125	0.343	0.153	0.387	0.282 **	0.465	0.080
	*p*									0.081	0.713	0.200	0.389	0.528	0.000	0.720	0.009	0.202	0.244	0.404	0.500	0.789	0.349	0.193	0.195	0.138	0.009	0.070	0.494
ALP	r									1	1	0.275	0.110	0.425	0.137	0.133	−0.020	0.193	0.023	0.413	0.047	0.396	0.086	0.353	0.054	−0.114	0.110	−0.041	0.106
	*p*											0.302	0.319	0.101	0.214	0.624	0.856	0.474	0.839	0.183	0.735	0.181	0.520	0.180	0.649	0.674	0.319	0.880	0.362
TC	r											1	1	0.232	0.365 **	0.369	0.350 **	0.933 **	0.903 **	−0.049	−0.154	0.357	−0.055	0.024	0.079	−0.454	0.040	−0.450	−0.008
	*p*													0.387	0.001	0.160	0.001	0.000	0.000	0.880	0.261	0.231	0.683	0.931	0.506	0.078	0.719	0.080	0.946
TG	r													1	1	−0.283	−0.271 *	0.334	0.373 **	0.315	0.125	−0.016	0.070	0.318	0.320 **	−0.230	0.081	−0.250	0.105
	*p*															0.288	0.013	0.206	0.001	0.319	0.365	0.957	0.601	0.229	0.006	0.392	0.464	0.350	0.365
HDL	r															1	1	0.220	0.180	0.119	−0.149	0.304	−0.072	0.148	0.071	−0.129	−0.086	−0.165	−0.098
	*p*																	0.413	0.103	0.712	0.276	0.313	0.592	0.585	0.551	0.635	0.437	0.541	0.402
LDL	r																	1	1	−0.028	−0.134	0.258	−0.060	−0.025	0.078	−0.425	0.024	−0.421	−0.065
	*p*																			0.931	0.334	0.394	0.659	0.−927	0.513	0.101	0.827	0.104	0.582
HOMA-IR	r																			1	1	0.839 **	0.913 **	0.738 **	0.218	0.291	0.060	0.434	0.027
	*p*																					0.001	0.000	0.006	0.114	0.359	0.666	0.159	0.857
Insulin	r																					1	1	0.218	0.043	0.099	0.045	0.225	−0.020
	*p*																							0.474	0.748	0.748	0.738	0.459	0.890
HbA1c	r																							1	1	0.156	0.302 **	0.177	0.323 **
	*p*																									0.565	0.010	0.512	0.009
FIB-4	r																									1	1	0.953 **	0.633 **
	*p*																											0.000	0.000
NAFLD	r																											1	1
	*p*																												

**Table 5:** The ROC curve results of miRNA expressions in the discrimination of MASLD patients compared to healthy individuals.

	AUC	*p*	95%CI	Cut-Off	Sensitivity	1-Spesifity
miR-99a	0.607	0.012	0.519–0.694	0.88	57	56
miR-146a	0.803	0.000	0.740–0.867	3.6	74	74
miR-640	0.615	0.007	0.532–0.698	1.06	59	60

**Table 6:** The ROC curve results of miRNA expressions in the discrimination of the hepatitis B vaccine response among MASLD patients.

	AUC	*p*	95%CI	Cut-Off	Sensitivity	1-Spesifity
miR-99a	0.520	0.800	0.364–0.676	1.2	50	50
miR-146a	0.489	0.888	0.329–0.649	6.27	43	42
miR-640	0.451	0.538	0.305–0.598	1.28	44	44

**Table 7:** The ROC curve results of miRNA expressions in the discrimination of hepatitis B vaccine response among healthy individuals.

	AUC	*p*	95%CI	Cut-Off	Sensitivity	1-Spesifity
miR-99a	0.995	0.000	0.986–1.000	1.24	97	96
miR-146a	0.822	0.000	0.733–0.911	1.72	69	69
miR-640	0.895	0.000	0.817–0.973	1.05	84	84

## Data Availability

All data from the manuscript will be available upon request by contacting Gamze Guney Eskiler.

## References

[1] Zhang J., Lin S., Jiang D., Li M., Chen Y., Li J., Fan J. (2020). Chronic hepatitis B and non-alcoholic fatty liver disease: Conspirators or competitors?. Liver Int..

[2] Global Viral Hepatitis. https://www.cdc.gov/hepatitis/global/index.html.

[3] Qiu S., He P., Fang X., Tong H., Lv J., Liu J., Zhang L., Zhai X., Wang L., Hu Z. (2018). Significant transcriptome and cytokine changes in hepatitis B vaccine non-responders revealed by genome-wide comparative analysis. Hum. Vaccin. Immunother..

[4] Zuckerman J.N. (2006). Protective efficacy, immunotherapeutic potential, and safety of hepatitis B vaccines. J. Med. Virol..

[5] Yang S., Tian G., Cui Y., Ding C., Deng M., Yu C., Xu K., Ren J., Yao J., Li Y. (2016). Factors influencing immunologic response to hepatitis B vaccine in adults. Sci. Rep..

[6] Walayat S., Ahmed Z., Martin D., Puli S., Cashman M., Dhillon S. (2015). Recent advances in vaccination of non-responders to standard dose hepatitis B virus vaccine. World J. Hepatol..

[7] Godkin A., Davenport M., Hill A.V. (2005). Molecular analysis of HLA class II associations with hepatitis B virus clearance and vaccine nonresponsiveness. Hepatology.

[8] Kumar R., Goh G.B.B. (2016). Chronic hepatitis B and fatty liver: Issues in clinical management. Clin. Res. Hepatol. Gastroenterol..

[9] Mehta M., Slaughter C., Xanthakos S.A., Kohli R.H. (2014). High Prevalence of Hepatitis B Non-Immunity in Pediatric Non Alcoholic Fatty Liver Disease Patients. Dig. Liver Dis..

[10] Joshi S.S., Davis R.P., Ma M.M., Tam E., Cooper C.L., Ramji A., Kelly E.M., Jayakumar S., Swain M.G., Jenne C.N. (2021). Reduced immune responses to hepatitis B primary vaccination in obese individuals with nonalcoholic fatty liver disease (NAFLD). npj Vaccines.

[11] Megahed F.A.K., Zhou X., Sun P., Marwa Mohamed E. (2021). The interplay between non-alcoholic fatty liver disease and innate immunity in hepatitis B virus patients. Egypt. Liver J..

[12] Wang B., Li W., Fang H., Zhou E. (2019). Hepatitis B virus infection is not associated with fatty liver disease: Evidence from a cohort study and functional analysis. Mol. Med. Rep..

[13] Appourchaux K., Dokmak S., Resche-Rigon M., Treton X., Lapalus M., Gattolliat C., Porchet E., Martinot-Peignoux M., Boyer N., Vidaud M. (2016). MicroRNA-based diagnostic tools for advanced fibrosis and cirrhosis in patients with chronic hepatitis B and C. Sci. Rep..

[14] Loureiro D., Tout I., Narguet S., Benazzouz S.M., Mansouri A., Asselah T. (2020). miRNAs as Potential Biomarkers for Viral Hepatitis B and C. Viruses.

[15] Wei Y.F., Cui G.Y., Ye P., Chen J.N., Diao H.Y. (2013). MicroRNAs may solve the mystery of chronic hepatitis B virus infection. World J. Gastroenterol..

[16] Xiong J., Zhang H., Wang Y., Wang A., Bian J., Huang H., Zheng Y., Sang X., Xu Y., Lu X. (2017). Hepatitis B virus infection and the risk of nonalcoholic fatty liver disease: A meta-analysis. Oncotarget.

[17] Chen H.Y., Tang N.H., Lin N., Chen Z.X., Wang X.Z. (2008). Hepatitis B virus X protein induces apoptosis and cell cycle deregulation through interfering with DNA repair and checkpoint responses. Hepatol. Res..

[18] Roni D.A., Pathapati R.M., Kumar A.S., Nihal L., Sridhar K., Tumkur Rajashekar S. (2013). Safety and efficacy of hepatitis B vaccination in cirrhosis of liver. Adv. Virol..

[19] Lin H.Y., Yang Y.L., Wang P.W., Wang F.S., Huang Y.H. (2020). The emerging role of microRNAs in NAFLD: Highlight of microRNA-29a in modulating oxidative stress, inflammation, and beyond. Cells.

[20] Zhang H., Li Q.Y., Guo Z.Z., Guan Y., Du J., Lu Y.Y., Hu Y.Y., Liu P., Huang S., Su S.B. (2012). Serum levels of microRNAs can specifically predict liver injury of chronic hepatitis B. World J. Gastroenterol..

[21] Wang S., Qiu L., Yan X., Jin W., Wang Y., Chen L., Wu E., Ye X., Gao G.F., Wang F. (2012). Loss of microRNA 122 expression in patients with hepatitis B enhances hepatitis B virus replication through cyclin G1-modulated P53 activity. Hepatology.

[22] Roderburg C., Urban G.W., Bettermann K., Vucur M., Zimmermann H., Schmidt S., Janssen J., Koppe C., Knolle P., Castoldi M. (2011). Micro-RNA profiling reveals a role for miR-29 in human and murine liver fibrosis. Hepatology.

[23] Ji F., Yang B., Peng X., Ding H., You H., Tien P. (2011). Circulating microRNAs in hepatitis B virus–infected patients. J. Viral Hepat..

[24] Hayes C.N., Akamatsu S., Tsuge M., Miki D., Akiyama R., Abe H., Ochi H., Hiraga N., Imamura M., Takahashi S. (2012). Hepatitis B virus-specific miRNAs and Argonaute2 play a role in the viral life cycle. PLoS ONE.

[25] Liu C.H., Ampuero J., Gil-Gómez A., Montero-Vallejo R., Rojas A., Munoz-Hernandez R., Gallego-Duran R., Romero-Gomez M. (2018). miRNAs in patients with non-alcoholic fatty liver disease: A systematic review and meta-analysis. J. Hepatol..

[26] Du J., Niu X., Wang Y., Wang R., Zhang Y., Zhao S., Nan Y. (2015). MiR-146a-5p suppresses activation and proliferation of hepatic stellate cells in nonalcoholic fibrosing steatohepatitis through directly targeting Wnt1 and Wnt5a. Sci. Rep..

[27] Fründt T., Krause L., Hussey E., Steinbach B., Köhler D., von Felden J., Schulze K., Lohse A.W., Wege H., Schwarzenbach H. (2021). Diagnostic and Prognostic Value of miR-16, miR-146a, miR-192 and miR-221 in Exosomes of Hepatocellular Carcinoma and Liver Cirrhosis Patients. Cancers.

[28] Bandiera S., Pernot S., El Saghire H., Durand S.C., Thumann C., Crouchet E., Ye T., Fofana I., Oudot M.A., Barths J. (2016). Hepatitis C Virus-Induced Upregulation of MicroRNA miR-146a-5p in Hepatocytes Promotes Viral Infection and Deregulates Metabolic Pathways Associated with Liver Disease Pathogenesis. J. Virol..

[29] Xiong Y., Chen S., Chen R., Lin W., Ni J. (2013). Association between microRNA polymorphisms and humoral immunity to hepatitis B vaccine. Hum. Vaccin. Immunother..

[30] Xiong Y., Chen S., Liu L., Zhao Y., Lin W., Ni J. (2013). Increased serum microRNA-155 level associated with nonresponsiveness to hepatitis B vaccine. Clin. Vaccine Immunol..

[31] Li D., Liu X., Lin L., Hou J., Li N., Wang C., Wang P., Zhang Q., Zhang P., Zhou W. (2011). MicroRNA-99a inhibits hepatocellular carcinoma growth and correlates with prognosis of patients with hepatocellular carcinoma. J. Biol. Chem..

[32] Celikbilek M., Baskol M., Taheri S., Deniz K., Dogan S., Zararsiz G., Gursoy S., Guven K., Ozbakır O., Dundar M. (2014). Circulating microRNAs in patients with non-alcoholic fatty liver disease. World J. Hepatol..

[33] Xiong F., Ma H., Qu Y., Wen F., Bao X., Han D., Lu J. (2016). Profiles of serum miR-99a, let-7c and miR-125b in hepatitis B virus (HBV)-associated chronic hepatitis, liver cirrhosis and hepatocellular carcinoma. Int. J. Clin. Exp. Pathol..

[34] Abdalla M.A., Haj-Ahmad Y. (2012). Promising Candidate Urinary MicroRNA Biomarkers for the Early Detection of Hepatocellular Carcinoma among High-Risk Hepatitis C Virus Egyptian Patients. J. Cancer.

[35] Wang G.X., Pan J.Y., Wang Y.J., Huang T.C., Li X.F. (2020). MiR-640 inhibition alleviates acute liver injury via regulating WNT signaling pathway and LRP1. Eur. Rev. Med. Pharmacol. Sci..

[36] Khan R.S., Bril F., Cusi K., Newsome P.N. (2019). Modulation of insulin resistance in nonalcoholic fatty liver disease. Hepatology.

[37] Kitade H., Chen G., Ni Y., Ota T. (2017). Nonalcoholic fatty liver disease and insulin resistance: New insights and potential new treatments. Nutrients.

